# Bone Formation and Anti-Inflammatory Properties of Iodine-Loaded Titanium Implants: An Animal Study

**DOI:** 10.3390/ma18214836

**Published:** 2025-10-22

**Authors:** Kazuto Yamada, Kazuya Inoue, Nanako Shimada, Tatsuya Kakutani, Yasuhisa Sawai, Naoko Imagawa-Fujimura, Kayoko Yamamoto, Nahoko Kato-Kogoe, Seiji Yamaguchi, Takaaki Ueno

**Affiliations:** 1 Department of Dentistry and Oral Surgery, Faculty of Medicine, Osaka Medical and Pharmaceutical University, 2-7 Daigakumachi, Takatsuki 569-8686, Japan; kazuto.yamada@ompu.ac.jp (K.Y.); nanako.shimada@ompu.ac.jp (N.S.); naoko.imagawa@ompu.ac.jp (N.I.-F.); kayoko.yamamoto@ompu.ac.jp (K.Y.); nahoko.kogoe@ompu.ac.jp (N.K.-K.); ueno@ompu.ac.jp (T.U.); 2 Osaka Yakin Kogyo Co., Ltd., Zuiko 4-4-28, Higashi Yodogawa-ku, Osaka 533-0005, Japan; kakutani@e.osakayakin.co.jp; 3 Department of Oral and Maxillofacial Surgery, Kanazawa Medical University, 1-1 Daigaku, Uchinada, Ishikawa 920-0293, Japan; y-sawai@kanazawa-med.ac.jp; 4 Department of Biomedical Sciences, College of Life and Health Sciences, Chubu University, 1200 Matsumoto-cho, Kasugai 487-8501, Japan; sy-esi@fsc.chubu.ac.jp

**Keywords:** anti-inflammatory, apatite-forming ability, iodine, micro/nano-surface structure, short dental Implant, surface treatment, titanium implant, TSH

## Abstract

Titanium implants are subjected to various surface treatments to improve their in vivo function. In this study, we evaluated the usefulness of titanium implants treated with acid, NaOH, CaCl_2_, heat, and ICl_3_ (Ac-NaCaThIo) in terms of in vivo bone-bonding strength, bone formation, and histological anti-inflammatory properties. Iodine-loaded experimental dental implants and commercial control dental implants were placed in rabbit femurs, and bone-bonding strength was evaluated by measuring the implant stability quotient (ISQ), bone formation using tissue specimens, and the effect of iodine using thyroid-stimulating hormone (TSH) levels. Iodine-loaded titanium plates and untreated titanium plates were placed on rat skulls and inoculated with *Streptococcus mitis* (*S. mitis*) solution to evaluate anti-inflammatory properties. Consequently, the experimental implants did not demonstrate non-inferiority in bone-bonding strength (ISQ) compared with the controls; however, histological specimens revealed dense bone contact and favorable bone formation. TSH levels showed no differences at 13 weeks, indicating no long-term adverse effects of iodine. The experimental tissue specimens of the soft tissue had fewer inflammatory cells than the control at 2 weeks after placement, demonstrating an anti-inflammatory effect. These results suggest that, although non-inferiority in ISQ was not demonstrated, Ac-NaCaThIo-treated implants showed favorable bone formation, dense bone contact, anti-inflammatory properties, and biosafety, indicating potential for future applications.

## 1. Introduction

Titanium is widely used in orthopedics and dentistry because of its high biocompatibility and corrosion resistance [1]. In the field of orthopedics, it is used in artificial joint replacement surgery and reconstruction surgery for bone defects with the aim of functional recovery. In the field of dentistry, it is used in reconstruction surgery for jawbone defects and in dental implant treatment for the aesthetic and functional recovery of missing teeth [2]. However, problems with treatment include implant loss due to poor bone-bonding and the need for early implant removal due to bacterial infection of the peri-implant tissues [3,4,5,6]. Various surface treatments have been used to solve these problems by improving the surface properties of the device such that it bonds to the surrounding bone at an early stage and imparts antibacterial properties to the device [7,8,9,10,11,12,13,14]. Surface treatment methods for titanium include hydroxyapatite plasma spraying, sandblasting, acid etching, anodic oxidation, and alkaline heat treatment; osseointegration between titanium and bone in vivo can be achieved by these surface treatment methods [7,8,9,10]. Additionally, titanium has been coated with antibacterial substances such as gentamicin or made to bind inorganic ions such as silver, iodine, gallium, and zinc, which are known to have antibacterial properties [11,12,13,14].

Among the various surface treatments, acid etching of titanium surfaces has been shown to produce a micro-scale structure on the surface and promote the proliferation and differentiation of osteoblast-like cells [15,16,17,18]. Oliveira et al. reported that titanium treated with acid etching followed by NaOH treatment formed a micro/nano-scale hierarchical structure on the surface, and that the effect of this surface structure was to enhance the expression of osteogenic markers such as Secreted Phosphoprotein 1 (SPP1), Runt-related transcription factor 2 (RUNX2), and Alkaline Phosphatase (ALP) compared with that of the micro-scale alone [19]. We also reported that by applying acid etching to titanium followed by NaOH-CaCl_2_-heated-ICl_3_ (NaCaThIo) treatment, a micro/nano-scale hierarchical structure is formed on the titanium surface, promoting cell proliferation, and improving early cell adhesion and bone differentiation potential compared to untreated titanium [20]. Furthermore, titanium and titanium alloys treated with NaCaThIo can be loaded with 0.7% to 10.5% iodine [21], and the iodine loaded on the titanium is slowly released, resulting in antibacterial effects against Methicillin-Resistant *Staphylococcus aureus* (MRSA), *Staphylococcus aureus* (*S. aureus*), *Escherichia coli* (*E. coli*), *Staphylococcus epidermidis* (*S. epidermidis*), and the oral bacteria *Streptococcus mitis* (*S. mitis*), and *Prevotella intermedia* (*P. intermedia*) [13,21,22].

By applying acid etching to titanium, followed by NaCaThIo (Ac-NaCaThIo) treatment, we believe it would be possible to develop a new biomaterial that induces bone formation and bone-bonding at an early stage and has antibacterial effects. Few studies have reported on in vivo bone formation and antibacterial evaluation of titanium implants treated with this method. Therefore, this study aimed to demonstrate the usefulness of Ac-NaCaThIo-treated implants in vivo. Titanium and bone have been reported to chemically bond in vivo when titanium has an apatite-forming ability in simulated body fluid (SBF) [23]. Thus, we evaluated the in vivo bone-bonding strength, bone formation, and biosafety of Ac-NaCaThIo-treated dental implants placed in rabbit femurs, and the in vivo anti-inflammatory properties of Ac-NaCaThIo-treated titanium plates placed in the parietal region of rats injected with bacterial solution.

## 2. Materials and Methods

### 2.1. In Vitro Apatite Formation, and In Vivo Bone-Bonding, Bone Formation, and Safety Evaluation

#### 2.1.1. *In Vitro* Evaluation of Apatite Formation Ability in SBF

**Figure 1:**
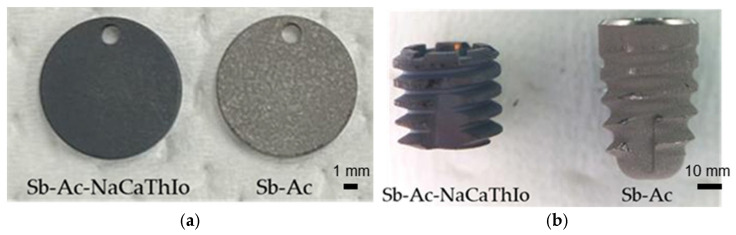
(**a**) Appearance of titanium disks for apatite formation evaluation. (**b**) Appearance of dental implants for bone-bonding strength, histological bone formation, and biosafety evaluation.

Pure JIS 4 type titanium disks with a diameter of 10 mm were used to evaluate apatite formation. The experimental group titanium disks were treated with alumina sandblasting, acid etching with oxalic acid, and NaCaThIo (Sb-Ac-NaCaThIo), and the control group titanium disks were treated with alumina sandblasting and acid etching with oxalic acid (Sb-Ac). NaCaThIo treatment is shown in Table 1. In the NaCaThIo process, samples were washed with ultrapure water, dried after each step, and finally sterilized with ethylene oxide gas. The surface treatment methods applied to the titanium disks and the appearance of the titanium disks are shown in Table 2 and Figure 1a, respectively.

The titanium disks of both groups were immersed in SBF for 3 and 7 d. After the titanium disks were removed from the SBF, their surfaces were coated with Pt/Pd, and apatite formation on the surfaces was observed under a field-emission scanning electron microscope (FE-SEM; JSM-IT710HR, JEOL, Ltd., Tokyo, Japan). Energy-dispersive X-ray spectroscopy (EDX; JSM-IT710HR, JEOL, Ltd., Tokyo, Japan) was performed to examine the constituent elements on their surfaces. The acceleration voltage was set to 15 kV.

#### 2.1.2. Placed Implants and Surface Preparation

Dental implants were used to evaluate bone-bonding strength, histological bone formation, and biosafety. Dental implants with a diameter of 4.4 mm and a height of 4.0 mm were developed as the experimental group, and the implant bodies were treated with Sb-Ac-NaCaThIo (Sb-Ac-NaCaThIo implants). Conversely, the control group was a commercial dental implant (Eight-Lobe Pro FB SAG PF 4.1–3.8 NC, Platon Japan Co., Ltd., Tokyo, Japan). The control implants with a diameter of 4.1 mm and height of 6.5 mm were treated with Sb-Ac (Sb-Ac implants). The surface treatment methods applied to the dental implants and the appearance of the dental implants are shown in Table 2 and Figure 1b, respectively.

#### 2.1.3. Surface Characterization of Placed Implants

The surfaces of Sb-Ac-NaCaThIo implants in the experimental group and Sb-Ac implants in the control group at the thread tops and valleys were observed under a field emission scanning electron microscope (FE-SEM; JSM-IT710HR, JEOL, Ltd., Tokyo, Japan) at an acceleration voltage of 15 kV. The three-dimensional surface properties of the implant surfaces were then determined using a confocal laser microscope (CLM; VK-X7000, KEYENCE Corp., Osaka, Japan), and the surface roughness values (arithmetic mean roughness, Ra; maximum roughness, Rz) were calculated from the surface roughness profiles at 21 arbitrary locations at the thread tops and valleys after correcting for the baseline. The Ra and Rz were expressed as the arithmetic mean ± standard deviation (SD).

#### 2.1.4. Experimental Animals and Implant Placement Order

The experimental protocol was reviewed and approved by the Animal Experiment Committee of the Graduate School of Medicine, Osaka Medical and Pharmaceutical University (approval number: AM23-109). Six 13-week-old New Zealand white rabbits were used as the subject animals, three of which were placed in the group with Sb-Ac-NaCaThIo implants (experimental group) and the remaining three animals were placed in the group with Sb-Ac implants (control group). 

General anesthesia was administered by intramuscular injection of 0.4 mL·kg^−1^ of an anesthetic solution mixed with ketamine hydrochloride and xylazine in equal volumes (corresponding to 10 mg·kg^−1^ ketamine hydrochloride and 4 mg·kg^−1^ xylazine), and maintenance anesthesia was administered with an isoflurane inhalation anesthetic solution. The hair was trimmed from the right and left knee joints to the femoral area, disinfected with povidone-iodine and ethanol solution, an incision was made to expose the femoral stem end, and six dental implants in each group were placed in the right and left rabbit femurs, one in each femur. After placement, the muscle, connective tissue, and skin were sutured with 3-0 nylon sutures (NescoSuture^®^, Alfresa Pharma Co., Osaka, Japan), and the surgical site was disinfected. 

The rabbits were intramuscularly administered 10 mg·kg^−1^ of enrofloxacin (Baytril^®^ 2.5% injectable solution, Elanco Japan K.K., Tokyo, Japan) for 3 days to prevent postoperative infection, and 20 µg·kg^−1^ of buprenorphine hydrochloride injection (buprenorphine injectable solution 0.2 mg, Nissin Pharmaceutical Co., Ltd., Yamagata, Japan) once a day for analgesia. 

#### 2.1.5. Bone-Bonding Strength Evaluation

Six dental implants (n = 6, two per rabbit) in the experimental and control groups were evaluated for bone-bonding strength at the time of placement and 13 weeks after placement by measuring the implant stability quotient (ISQ) using resonance frequency analysis (RFA) with an implant stability analysis device (Osstell IDx, Osstell AB, Gothenburg, Sweden). The ISQ values were expressed as the arithmetic mean ± standard deviation (SD). In this study, these values that showed abnormally low readings were considered clinically implausible or likely to represent measurement errors. These values were further evaluated using Tukey’s fences and treated as outliers in the analysis.

#### 2.1.6. Histological Bone Formation Evaluation 

After euthanasia by an intravenous overdose (>50 mg·kg^−1^) of injectable sodium thiamylal, three of six placed dental implants in each group were removed together with the surrounding bone as a single block. The excised tissue samples were fixed in 10% neutral buffered formalin solution, followed by dehydration and degreasing with ethanol and acetone. Tissue samples were embedded in MMA and then sectioned using a micro-cutting machine (BS-300CP/EXAKT^®^, Meiwafosis Co. Ltd., Tokyo, Japan) and a micro-grinding machine (MG-400CS/EXAKT^®^, Meiwafosis Co. Ltd., Tokyo, Japan). Subsequently, ground sections approximately 60–80 μm thick were stained with toluidine blue. After staining, histological evaluation was performed using an optical microscope (Nikon ECLIPSE Ci^®^, Nikon, Tokyo, Japan). Histological parameters were measured using image analysis software (ImageJ version 1.54g, National Institutes of Health, Bethesda, MD, USA). Specimens were coded to blind the examiner to group allocation. Each measurement was repeated twice, and the mean value was used as the final result. Intra-rater repeatability was confirmed.

a. Bone area occupancy (BAO)

Two regions of interest (ROI) bound by any contiguous thread top and valley in the bone were selected on the tissue specimen, and the bone area fraction occupancy (BAO) of these regions was calculated. BAO was calculated using the following formula:

(1) $$BAO\left(\%\right)=\frac{B}{A}\;\times 100$$

A indicates the area of ROI bounded by any contiguous thread top and valley. B indicates the area of bone tissue present in the ROI.

b. Bone-implant contact ratio (BIC)

The bone-implant contact ratio (BIC) was calculated for the two sites where BAO was measured. BIC was calculated using the following formula:

(2) $$BIC\left(\%\right)=\frac{D}{C}\;\times 100$$

C indicates the circumferential length between the threads in the area where BAO was measured. D indicates the length of direct contact between the bone tissue and the implant body in the area where the BAO was measured.

#### 2.1.7. Biosafety Evaluation by Blood Collection 

Blood samples (1.5 mL) were collected at weeks 1, 4, 12, and 13 after placement from both groups, and thyroid-stimulating hormone (TSH) levels in the blood were measured using a Thyrotropin/TSH ELISA Kit (LS-F67417-1, LifeSpan Biosciences Inc., Seattle, WA, USA). At weeks 1, 4, and 12, the rabbits were held unanesthetized, and blood was collected from the auricular vein, and at week 13, blood was collected from the heart under anesthesia. Blood samples were stored in Sepaclean A containing a serum separator, and the serum was separated using a refrigerated centrifuge (Model 5922, Kubota Manufacturing Co., Ltd., Tokyo, Japan) or a high-speed refrigerated centrifuge (Model 6200, Kubota Manufacturing Co., Ltd., Tokyo, Japan). The separated serum was stored in an ultra-low temperature freezer (CLN-52UD1, Nihon Freezer Co., Ltd., Tokyo, Japan) at temperatures below −60 °C. The TSH values were expressed as the arithmetic mean ± standard deviation (SD). 

### 2.2. In Vivo Anti-Inflammatory Evaluation 

#### 2.2.1. Placement Materials and Surface Preparation

**Figure 2:**
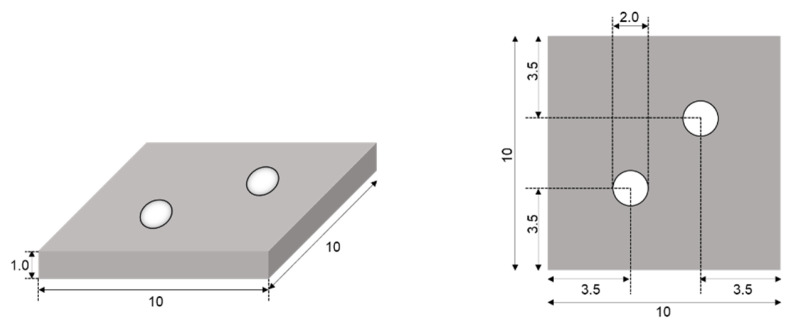
Schematic diagram of the placed titanium plate used for anti-inflammatory evaluation (length unit is mm).

JIS Class 2 pure titanium plates were used as the placement materials. Titanium plates (10 mm × 10 mm × 1 mm) were prepared, as shown in Figure 2, and small holes for screw fastening were made in the center of the titanium plates. The surface treatment methods applied to the titanium plates are shown in Table 3. In the experimental group, the titanium plates were surface-treated with Ac-NaCaThIo. For the acid etching treatment, a mixed acid solution consisting of 66.3% sulfuric acid solution and 10.6% hydrochloric acid solution at a weight ratio of 1:1 was used. After each treatment, the surfaces were washed with ultrapure water, dried, and finally sterilized with ethylene oxide gas. Titanium plates without surface treatment were used as controls.

#### 2.2.2. Preparation of Bacterial Solution

*Streptococcus mitis* (*S. mitis*, ATCC 49456), an oral bacterium, was used in this study. *S. mitis* was cultured at 37 °C in 10 mL of Brain Heart Infusion (BHI) broth (ATCC Medium 44), prepared from BHI Broth (37.0 g; Becton Dickinson, NJ, USA; Cat. No. 237500) dissolved in 1000 mL of deionized water. The final bacterial concentration was prepared by diluting with BHI broth to an OD value of 0.6 using a Cell Density Meter (CO8000 Biowave Cell Density Meter, Biochrom Ltd., Cambridge, UK).

#### 2.2.3. Experimental Animals and Titanium Plate Placement Order

**Figure 3:**
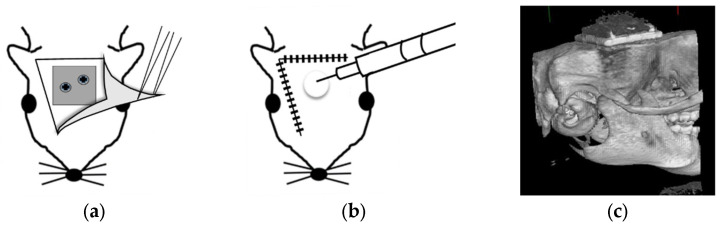
Schematic diagram of the experimental procedure for anti-inflammatory evaluation. (**a**) Titanium plate placement. (**b**) Suturing the incision and injection of *S. mitis* solution into the rat’s parietal region. (**c**) Micro-CT image of a rat with a titanium plate.

The experimental protocol was reviewed and approved by the Animal Experiment Committee of the Graduate School of Medicine at Osaka Medical and Pharmaceutical University (approval number AM24-026). A schematic of the protocol is shown in Figure 3.

Thirty-six 12-week-old male Sprague–Dawley rats were used in this study. The rats were divided into four groups as follows: (1) Ac-NaCaThIo-treated titanium plate placed on rat skull and *S. mitis* solution inoculation group (Ac-NaCaThIo + *S. mitis* group), (2) untreated titanium plate placed on rat skull and *S. mitis* solution inoculation group (no treatment + *S. mitis* group), (3) Ac-NaCaThIo-treated titanium plates were placed on rat skull and *S. mitis* solution was not inoculated (Ac-NaCaThIo only group), and (4) untreated titanium plates were placed on rat skull and *S. mitis* solution was not inoculated (no treatment only group). Three rats were used in each group, and a titanium plate was placed for 1, 2, or 4 weeks in all groups.

The rats were sedated with an isoflurane inhalation anesthetic solution, and after the hair was trimmed, the rats were disinfected with povidone-iodine and ethanol solution, and the parietal region of the rat was locally anesthetized with 2% xylocaine. An incision was made in the parietal skin, and a mucoperiosteal flap was created. The skull was then exposed, and the titanium plate of each group was fixed to the rat’s skull using a micro screw (max drive screw, KLS Martin Japan, Tokyo, Japan) with a diameter of 1.5 mm and a length of 4.0 mm. The periosteum was sutured with 5-0 polyglactin suture (Vicryl Plus^®^, Johnson & Johnson K.K., Tokyo, Japan), and the skin with 3-0 silk suture (Sofsilks^®^ VS872-2, Covidien Japan K.K., Tokyo, Japan). After suturing, 100 µL of *S. mitis* solution, adjusted to an OD value of 0.6, was injected into the parietal area of rats in inoculated groups (1) and (2) from the skin on the top of the head toward the placed titanium plate.

#### 2.2.4. Histological Anti-Inflammatory Evaluation

After euthanasia by inhalation overdose of isoflurane, an incision was made according to the incision line at the time of placement, and a mucoperiosteal flap was raised again. After exposure to the titanium plate, the parietal soft tissue in contact with it was removed. The excised tissue samples were fixed in 10% neutral buffered formalin solution, followed by dehydration and degreasing with ethanol and acetone. After embedding the tissue samples in paraffin, thin-sectioned specimens with a thickness of 3 μm were prepared using a microtome (REM-710; Yamato Kohki Industrial, Saitama, Japan). The thin-section specimens were subjected to hematoxylin and eosin (HE) staining. After staining, histological evaluation was performed using an optical microscope (Nikon ECLIPSE Ci^®^, Nikon, Tokyo, Japan). Specimens were coded to blind the evaluators to group allocation, and all histological evaluations were performed accordingly. The evaluation was performed by three independent evaluators using a set-score system. The scores were as follows: score 0, no inflammatory cell infiltration; score 1, mild inflammatory cell infiltration, localized; score 2, moderate inflammatory cell infiltration; score 3, severe inflammatory cell infiltration. The scores at 1, 2, and 4 weeks in each group were summarized as means, and the changes in scores among the groups were compared and evaluated. To confirm the consistency among the three evaluators, inter-rater reliability of the inflammation scores was assessed using Fleiss’ kappa (κ).

### 2.3. Statistical Analysis

Continuous variables (Ra, Rz, ISQ, BAO, BIC, and TSH) were expressed as the mean ± standard deviation (SD). Histological inflammation scores were summarized as mean values. Statistical analyses were performed using JMP^®^ 18 (JMP Statistical Discovery LLC., Cary, NC, USA).

For the bone integration outcomes (ISQ, BAO, and BIC), non-inferiority was first assessed by verifying that the lower bound of the one-sided 97.5% confidence interval for the difference between groups exceeded the prespecified non-inferiority margin (Δ). The margins were set at ±5 ISQ units for ISQ and ±10 percentage points for BAO and BIC, based on previous reports regarding measurement reproducibility and clinically meaningful differences in implant stability and histomorphometric analyses [24,25,26,27]. If non-inferiority was demonstrated, superiority was subsequently tested using the Mann–Whitney U test (two-sided). Effect sizes were reported as Hodges–Lehmann median differences with 95% CIs.

Surface roughness values (Ra, Rz) and serum TSH values were compared between groups using the Mann–Whitney U test (two-sided). A two-sided significance level of *p* < 0.05 was considered statistically significant unless otherwise specified.

## 3. Results

### 3.1. In Vitro Apatite Formation, and In Vivo Bone-Bonding, Bone Formation, and Safety Evaluation

#### 3.1.1. *In Vitro* Evaluation of Apatite Formation Ability

**Figure 4:**
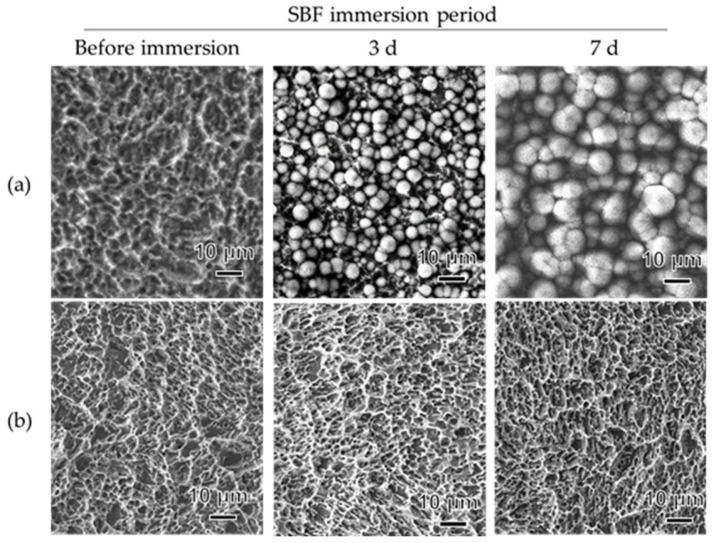
FE-SEM images of the placed titanium disk surface before, 3 d after, and 7 d after immersion in SBF. (**a**) Titanium disk with Sb-Ac-NaCaThIo treatment. (**b**) Titanium disk with Sb-Ac treatment.

**Figure 5:**
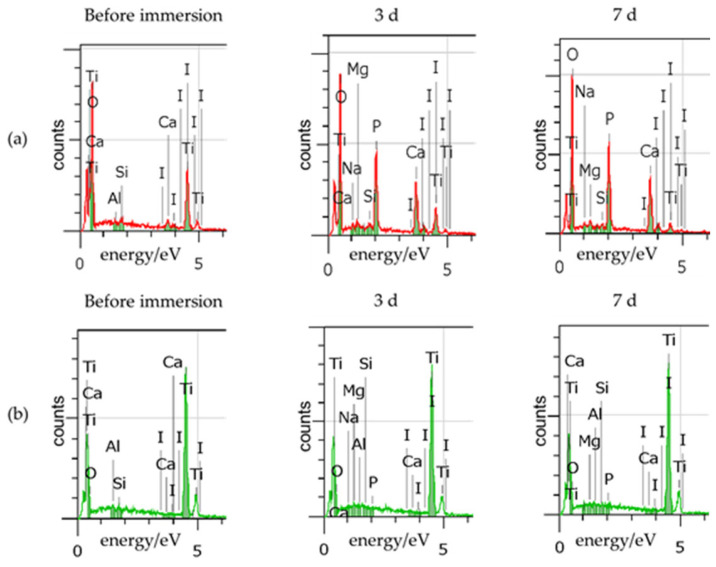
EDX analysis profile of the placed titanium disk surface. (**a**) Titanium disk with Sb-Ac-NaCaThIo treatment. (**b**) Titanium disk with Sb-Ac treatment.

SEM images of the Sb-Ac-NaCaThIo-treated titanium disks of the experimental group and Sb-Ac-treated titanium disks of the control group before, 3 d after, and 7 d after immersion in SBF are shown in Figure 4. Apatite-like spherical structures were formed on the surface of the Sb-Ac-NaCaThIo-treated titanium disks after 3 and 7 d of immersion in SBF. The results of the EDX analysis of the surfaces of the Sb-Ac-NaCaThIo-treated titanium disks and Sb-Ac-treated titanium disks after 3 and 7 d of SBF immersion are shown in Figure 5 and Table 4. After 3 and 7 d of SBF immersion, large amounts of Ca and P, which are components of apatite, were detected in the Sb-Ac-NaCaThIo-treated titanium disks after immersion in SBF. In contrast, no Ca or P was detected in the Sb-Ac-treated titanium disks.

#### 3.1.2. Surface Characterization of Placement Implants

**Figure 6:**
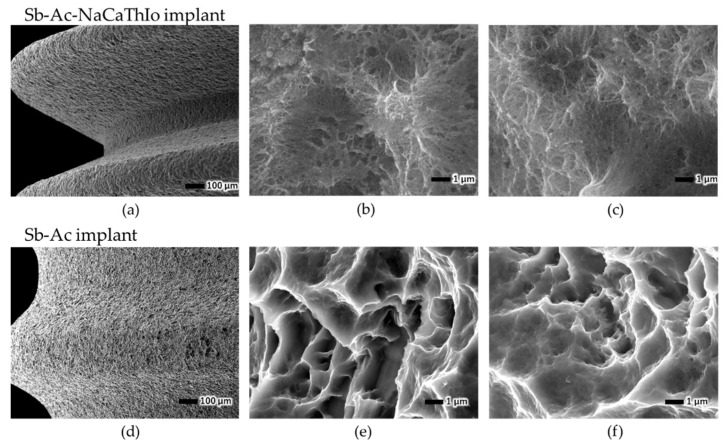
FE-SEM images of the placed implant surface. Images (**a**–**c**) show Sb-Ac-NaCaThIo implant, and (**d**–**f**) show Sb-Ac implant. (**a**) Thread tops and valleys. (**b**) Thread tops. (**c**) Thread valleys. (**d**) Thread tops and valleys. (**e**) Thread tops. (**f**) Thread valleys. Images were obtained at an accelerating voltage of 15 kV.

**Figure 7:**
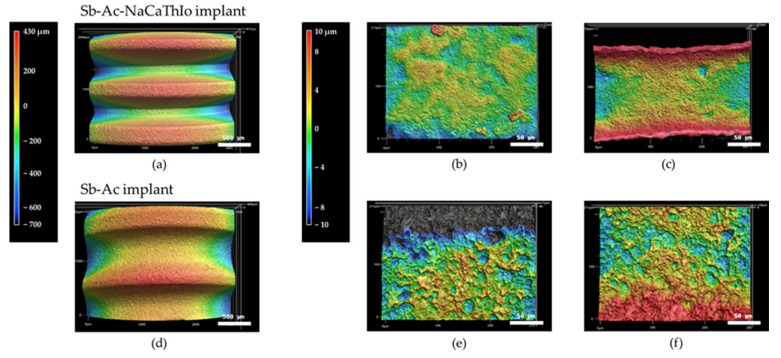
3D CLM images of the placed implant surface. Images (**a**–**c**) show Sb-Ac-NaCaThIo implant, and (**d**–**f**) show Sb-Ac implant. (**a**) Thread tops and valleys. (**b**) Thread tops. (**c**) Thread valleys. (**d**) Thread tops and valleys. (**e**) Thread tops. (**f**) Thread valleys.

FE-SEM and 3D CLM images of the surfaces of the thread tops and valleys in the experimental Sb-Ac-NaCaThIo and control Sb-Ac implants are shown in Figure 6 and Figure 7. In the experimental Sb-Ac-NaCaThIo implants, micro/nano-scale hierarchical structures were observed at the thread tops and valleys, as shown in Figure 6b,c. In contrast, the control Sb-Ac implants exhibited only micro-scale structures at the thread tops and valleys, as shown in Figure 6e,f. Similarly, in the 3D CLM images, the experimental Sb-Ac-NaCaThIo implants displayed relatively uniform micro/nano-scale hierarchical structures at the thread tops and valleys, as shown in Figure 7b,c, whereas the control Sb-Ac implants showed irregular micro-scale roughness with larger peak-to-valley distances at the thread tops and valleys, as shown in Figure 7e,f.

**Figure 8:**
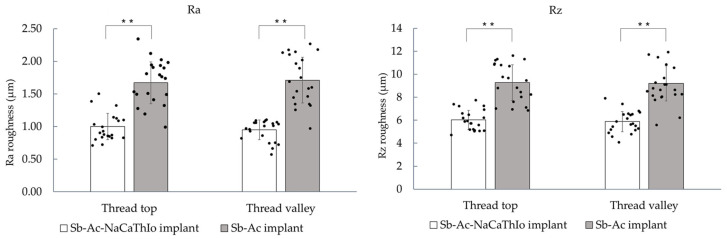
Arithmetic mean roughness (Ra) and maximum roughness (Rz) of the thread tops and valleys of Sb-Ac-NaCaThIo and Sb-Ac implants. Analytic unit: measurement site (n = 21 per location per group). Data are presented as mean ± SD (bars), with individual data points (dots) overlaid. Statistical analysis: Mann–Whitney U test (two-sided). * *p* < 0.05; ** *p* < 0.01; n.s., not significant.

The Ra and Rz values of both implants are shown in Figure 8. The Ra values of the thread tops and valleys in the experimental Sb-Ac-NaCaThIo implants and the control Sb-Ac implants were 1.00 ± 0.02 µm, 0.95 ± 0.15 µm, and 1.67 ± 0.32 µm, 1.71 ± 0.35 µm, respectively. The Rz values of the thread tops and valleys in the experimental Sb-Ac-NaCaThIo implants and the control Sb-Ac implants were 6.02 ± 0.84 µm, 5.88 ± 0.88 µm, and 9.28 ± 1.55 µm, 9.21 ± 1.53 µm, respectively. Both Ra and Rz values of the thread tops and valleys were significantly higher in the control implants than in the experimental implants (all *p* < 0.00001).

#### 3.1.3. Bone-Bonding Strength Evaluation

**Figure 9:**
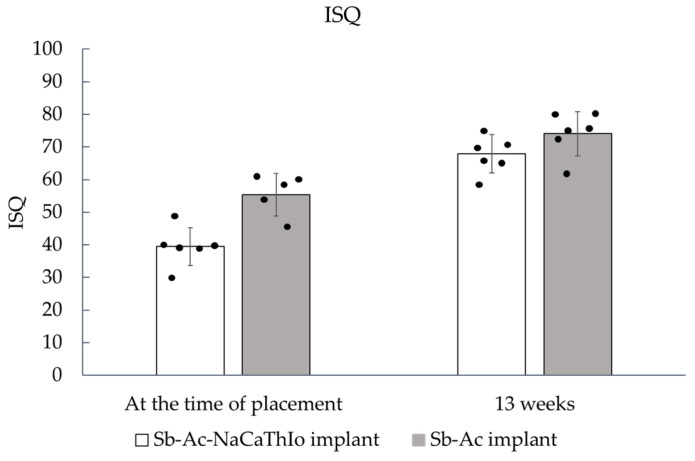
ISQ values at placement and 13 weeks after placement for Sb-Ac-NaCaThIo and Sb-Ac implants. The analytic unit was the implant (n = 6 per group, except Sb-Ac at placement, where n = 5). Data are presented as mean ± SD (bars), with individual data points (dots) overlaid.

The ISQ values at the time of placement and 13 weeks after placement are shown in Figure 9. At placement, one Sb-Ac implant showed an abnormally low ISQ value of 10. Based on Tukey’s fences, this value was judged as an outlier and excluded from the analysis. As a result, the number of Sb-Ac implants analyzed at placement was n = 5. 

**Figure 10:**
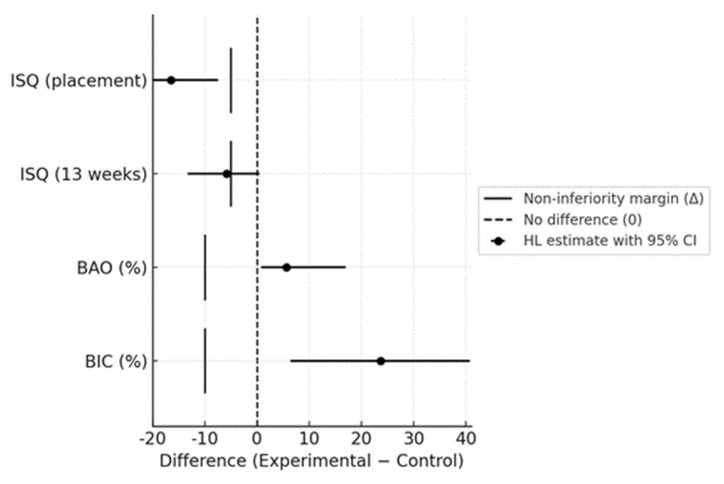
Non-inferiority analysis of ISQ, BAO, and BIC. Points represent Hodges–Lehmann median differences (experimental − control), and horizontal bars indicate 95% confidence intervals. The dashed vertical line represents no difference (0), and the solid vertical bars represent non-inferiority margins (Δ = −5 ISQ units for ISQ at placement and 13 weeks; Δ = −10% for BAO and BIC).

The ISQ values at placement and at 13 weeks, and the results of the non-inferiority analysis, are shown in Figure 9 and Figure 10, respectively. The ISQ values of the experimental Sb-Ac-NaCaThIo implants and the control Sb-Ac implants at placement were 39.5 ± 5.8 and 55.4 ± 6.5, respectively. The Hodges–Lehmann estimated difference (experimental − control) was −16.5; 95% CI, −22.5 to −7.5. The lower bound of the CI did not exceed the prespecified non-inferiority margin (Δ = −5 ISQ units), and non-inferiority was therefore not demonstrated.

At 13 weeks, the ISQ values of the Sb-Ac-NaCaThIo implants and the Sb-Ac implants were 67.9 ± 5.9 and 74.1 ± 6.8, respectively. The Hodges–Lehmann estimated difference was −5.8 (95% CI: −13.3 to 0.5). Similarly, the lower bound of the CI did not exceed the prespecified non-inferiority margin (Δ = −5), and non-inferiority was not demonstrated.

#### 3.1.4. Histological Bone Formation Evaluation

**Figure 11:**
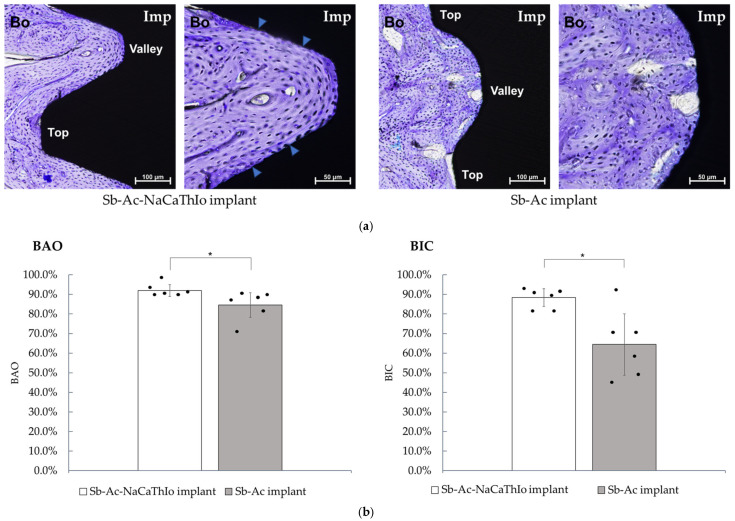
(**a**) Histological images of Sb-Ac-NaCaThIo and Sb-Ac implants placed in rabbit femurs 13 weeks after placement. Imp: dental implant, Bo: rabbit femur, Top: thread top, Valley: thread valley. (**b**) Bone area occupancy (BAO) and bone-implant contact ratio (BIC) in the regions enclosed by thread tops and valleys. Analytic unit: ROI on dental implant sections (n = 6 per group). Data are presented as mean ± SD (bars), with individual data points (dots) overlaid. Statistical analysis: Mann–Whitney U test (two-sided). * *p* < 0.05; n.s., not significant.

Representative tissue images of both implants are shown in Figure 11a. Both implants showed good bone formation in the area bounded by the thread tops and valleys, but the experimental Sb-Ac-NaCaThIo implants showed bone formation with almost no voids in the ROI. In the Sb-Ac-NaCaThIo implants, as indicated by the blue arrow, dense contact between the newly formed bone and the implant was observed.

- Bone area occupancy (BAO)

As shown in Figure 11b, the BAO of the experimental Sb-Ac-NaCaThIo implants and the control Sb-Ac implants were 92.0 ± 3.0% and 84.6 ± 6.3%, respectively. As shown in Figure 10, the Hodges–Lehmann estimated difference (experimental − control) was +5.6% (95% CI: +0.8 to +17.0). The lower bound of the CI exceeded the non-inferiority margin (Δ = −10%), demonstrating non-inferiority. Superiority was subsequently tested by the Mann–Whitney U test (*p* = 0.03).

b. Bone-implant contact ratio (BIC)

As shown in Figure 11b, the BIC of the experimental Sb-Ac-NaCaThIo implants and the control Sb-Ac implants were 88.4 ± 4.5% and 64.4 ± 15.7%, respectively. As shown in Figure 10, the Hodges–Lehmann estimated difference was +23.7% (95% CI: +6.4 to +40.7). The lower bound exceeded the non-inferiority margin (Δ = −10%), demonstrating non-inferiority, and superiority was subsequently tested (*p* = 0.04).

#### 3.1.5. Blood Evaluation

**Figure 12:**
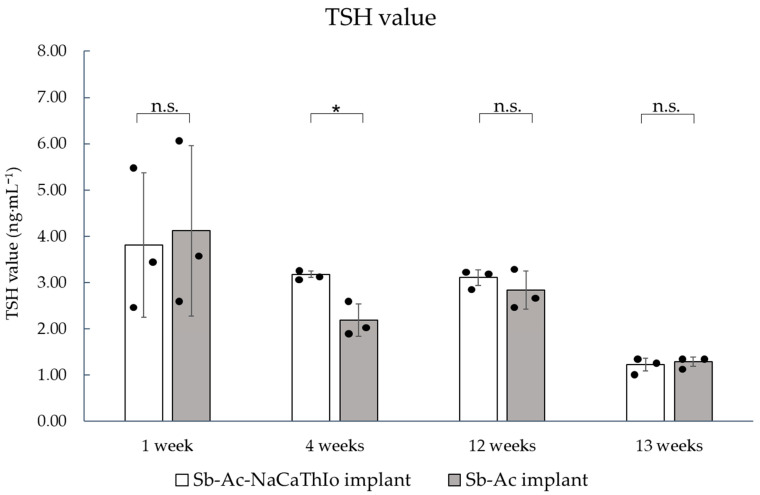
Serum TSH levels at 1, 4, 12, and 13 weeks after implant placement. Analytic unit: rabbit (n = 3 per group per time point). Data are presented as mean ± SD (bars), with individual data points (dots) overlaid. Statistical analysis: Mann–Whitney U test (two-sided). * *p* < 0.05; n.s., not significant.

The TSH levels at 1, 4, 12, and 13 weeks after placement are shown in Figure 12. After 1 week of placement, the TSH levels of the experimental Sb-Ac-NaCaThIo implants and the control Sb-Ac implants were 3.81 ± 1.56 ng·mL^−1^ and 4.12 ± 1.83 ng·mL^−1^, respectively. However, no significant difference was noted between the two implants (*p* = 0.83). After 4 weeks of placement, the TSH levels of the experimental Sb-Ac-NaCaThIo implants and the control Sb-Ac implants were 3.18 ± 0.07 ng·mL^−1^ and 2.18 ± 0.35 ng·mL^−1^, respectively. The Sb-Ac-NaCaThIo implants showed significantly higher values than the Sb-Ac implants (*p* = 0.01). After 12 weeks of placement, the TSH levels of the experimental Sb-Ac-NaCaThIo implants and the control Sb-Ac implants were 3.11 ± 0.17 ng·mL^−1^ and 2.84 ± 0.42 ng·mL^−1^, respectively. No significant change was noted in TSH levels compared to 4 weeks after placement, and no significant difference was found between the two implants (*p* = 0.35). After 13 weeks of placement, the TSH levels of the experimental Sb-Ac-NaCaThIo implants and the control Sb-Ac implants were 1.23 ± 0.14 ng·mL^−1^ and 1.29 ± 0.10 ng·mL^−1^, respectively. The TSH levels decreased compared to those after 12 weeks of placement, and no significant difference was noted in TSH values between the Sb-Ac-NaCaThIo and Sb-Ac implants (*p* = 0.55). 

### 3.2. In Vivo Anti-Inflammatory Evaluation

#### Histological Anti-Inflammatory Evaluation

**Figure 13:**
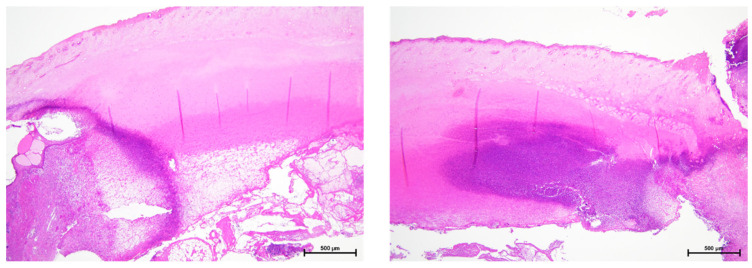
Representative two images of specimens showing significant inflammatory cell infiltration.

**Figure 14:**
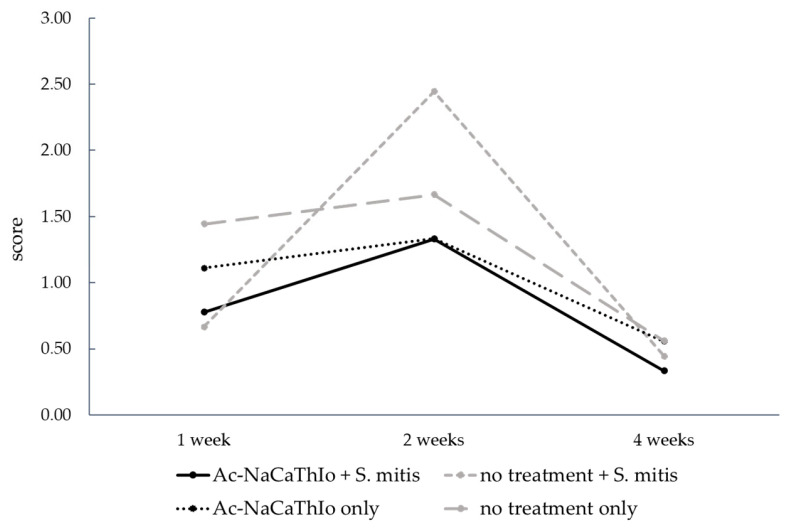
Changes in mean inflammation scores over time in each group. Analytic unit: rat (n = 3 per group). Scores (0–3) were evaluated on HE-stained cranial sections. Data are presented as mean values (lines with markers).

The representative images of tissue specimens showing significant inflammatory cell infiltration are depicted in Figure 13. In the tissue images, inflammatory cell infiltration was observed in all groups at 1 and 2 weeks; however, more significant inflammatory cell infiltration was observed at 2 weeks. In addition, at 4 weeks, the degree of inflammatory cell infiltration was significantly reduced in all the groups. The change in mean scores over time is shown in Figure 14. In the score evaluation, the score values were highest in all groups at two weeks after placement, and were highest in the (2) no treatment + *S. mitis* group at two weeks after placement. The inter-rater reliability of the inflammation scores among the three evaluators showed substantial agreement (Fleiss’ κ = 0.53). 

## 4. Discussion

This study aimed to demonstrate the usefulness of implants treated with Ac-NaCaThIo in terms of apatite formation in vitro, and bone-bonding strength, bone formation, the presence or absence of the harmful effects of iodine, and anti-inflammatory properties in vivo. First, the apatite formation ability in SBF was evaluated using titanium disks that had been treated with Sb-Ac-NaCaThIo as an experimental group and Sb-Ac as a control group. We previously reported that Ac-NaCaThIo-treated titanium plates have excellent apatite-forming ability [20]; however, in the current study, acid treatment was performed using a solution different from that in the previous experiment, warranting verification. In SBF, the experimental Sb-Ac-NaCaThIo-treated titanium disks formed apatite-like spherical structures within 3 days, whereas the control Sb-Ac-treated titanium disks did not, even after 7 days. Apatite is generally expressed using the chemical formula M_10_(ZO_4_)_6_ X_2_, where M is substituted by Ca, Sr, Ba, Ra, Na, K, Pb, or Cd; Z by P, As, V, or Si; and X by OH, Cl, F, Br, or I. In this study, large amounts of Ca and P ions were detected in the experimental titanium plates using EDX, suggesting that the spherical structure observed using SEM was apatite. In addition, it has been reported that titanium chemically bonds with bone when it has the apatite-forming ability in SBF [23]. Our study results indicate that Sb-Ac-NaCaThIo implants are expected to have good bone-bonding strength. Fujibayashi et al. proposed the practical use of materials that can form apatite in SBF within 3 days [28]. Therefore, we decided to evaluate bone formation and bone- bonding strength in vivo by using Sb-Ac-NaCaThIo-treated implants.

Next, the surface properties of the implants that had been treated with these surface treatments were evaluated. The surface properties of implants are important factors influencing cellular activity and apatite formation [29,30], and micro-scale surfaces improve the initial stability of implants, whereas nano-scale surfaces improve cell adhesion and differentiation [21,30,31]. As a result, micro/nano-scale hierarchical structures were observed on the surfaces of thread tops and valleys in the Sb-Ac-NaCaThIo implants, and micro-scale structures were observed on thread tops and valleys in the Sb-Ac implants. Since nano-scale structures are produced by NaCaThIo treatment [19], it was found that the nano-scale structures observed only in the implants of the experimental group were produced by NaCaThIo treatment. Furthermore, materials with micro/nano-scale hierarchical structures have been reported to enhance the activity of the surrounding osteoblasts compared to those with micro-scale structures [19]. Based on these reports, it can be expected that Sb-Ac-NaCaThIo implants will have stronger bone-bonding strength at an early stage because of increased activation of the surrounding osteoblasts. A comparison of the surface roughness between the two groups showed significantly higher values for the Sb-Ac implants at the top and the valley of the threads. As mentioned above, the surfaces of the Sb-Ac-NaCaThIo implants showed a micro/nano-scale hierarchical structure, and those of the Sb-Ac implants showed a micro-scale structure. The micro/nano-scale hierarchical structure inevitably reduces the surface roughness because the valley distance from the reference point is shorter. This is consistent with the results of other literature [20], confirming that the surface roughness results in this study are valid. 

In this study, surface-treated implants were placed in animals and evaluated in vivo. The experimental Sb-Ac-NaCaThIo implants were designed with a shorter length of 4.0 mm to examine the effectiveness of the surface treatment, with a diameter of 4.4 mm determined by mechanical strength requirements. Conversely, the control Sb-Ac implants selected for comparison had a diameter of 4.1 mm and a length of 6.5 mm, and were chosen because their surface area was 88 mm^2^, which was close to the 98 mm^2^ of the experimental implants.

The success rate of implants largely depends on osseointegration. Various methods have been used to evaluate implant stability as an indicator of osseointegration. In this study, we measured the ISQ using resonance frequency analysis (RFA), a non-invasive method that has been reported to be clinically effective [32,33,34]. ISQ is expressed on a scale from 1 to 100, with higher values indicating greater stability [35]. In general, ISQ values above 70 indicate high stability, values of 60–69 indicate moderate stability, and values below 60 indicate low stability [35]. 

In the present experiment, an abnormally low ISQ value was observed in one Sb-Ac implant at placement, which was excluded as an outlier based on Tukey’s fences. This value was unlikely to reflect actual implant stability and was most likely attributable to technical factors such as insufficient fixation of the SmartPeg or an improper probing angle. Importantly, exclusion of this outlier did not affect the overall interpretation of the results.

At placement, the non-inferiority analysis of ISQ values did not meet the prespecified margin (Δ = ±5 ISQ), indicating that non-inferiority could not be confirmed. Primary stability depends largely on the mechanical engagement between the implant and cortical bone [36]. Although no clear correlation has been reported between ISQ values and implant diameter [37,38,39,40], several studies have demonstrated an association between ISQ values and implant length [41,42]. Farronato et al. reported that RFA measurements are influenced by implant length [43], and Silva et al. showed a moderate positive correlation between ISQ values and implant length for 4.0 mm and 6.0 mm implants [44]. Therefore, the lack of non-inferiority at placement was likely attributable to the difference in implant length. At 13 weeks, the results did not exceed the margin; thus, non-inferiority was not demonstrated. Although no correlation between ISQ values and implant diameter has been reported in the evaluation of secondary stability [38,40,45], the potential influence of implant length cannot be excluded. Thus, the absence of non-inferiority at 13 weeks may also be explained by the difference in implant length. On the other hand, materials with micro/nano-hierarchical structures have been reported to enhance osteoblast activity compared with micro-scale structures [19]. The increase in ISQ values observed in the experimental Sb-Ac-NaCaThIo implants is therefore considered to reflect the activation of osteoblasts induced by the micro/nano-hierarchical surface. 

In the histological evaluation of bone formation, the experimental Sb-Ac-NaCaThIo implants showed good bone formation within the ROI and dense bone-to-implant contact. Furthermore, quantitative assessments demonstrated non-inferiority for both BAO and BIC, with the lower bounds of the confidence intervals exceeding the prespecified margin (Δ = ±10%). In addition, superiority testing revealed significantly higher values for the Sb-Ac-NaCaThIo implants compared with the controls. These results clearly indicate that the Ac-NaCaThIo treatment provided greater efficacy in promoting bone formation and bone-to-implant contact than the conventional treatment.

Previous studies have reported that micro/nano-hierarchical structures enhance osteoblast activity compared with micro-scale structures [19], and Johansson et al. showed that implants with such hierarchical surfaces exhibited favorable values for BIC, BAO, and removal torque, thereby demonstrating superior osseointegration properties [46]. The higher BAO and BIC observed in the present study are therefore considered to reflect these biological effects.

The difference in thread geometry between the Sb-Ac-NaCaThIo implants and the Sb-Ac implants also requires consideration. Kuroshima et al. reported that implant design influenced BAO and BIC under loading conditions but had little effect under non-loading conditions [47]. As the present study was conducted under non-loading conditions, the difference in thread geometry was unlikely to have had a major impact on the outcomes.

Blood evaluation was performed to assess the effect of iodine released from the experimental implants. Because most iodine in the blood is protein-bound as thyroxine (T4) and triiodothyronine (T3), direct quantification is difficult. Thyroid function is regulated by the pituitary hormone TSH, which increases in response to reduced thyroid hormones and is suppressed when hormone levels rise. Therefore, thyroid function was evaluated indirectly by measuring TSH, a sensitive and widely accepted marker of thyroidal feedback regulation [48].

At 1 week after placement, TSH levels were elevated in both Sb-Ac-NaCaThIo and Sb-Ac implants, likely reflecting surgical trauma and foreign body reaction. At 4 weeks, TSH levels declined in the Sb-Ac implants as surgical stress resolved, whereas persistently higher TSH values in the Sb-Ac-NaCaThIo implants indicated a transient impairment of thyroid function due to continuous iodine release [49]. Previous studies have shown that NaCaThIo treatment enables stable iodine loading and its gradual release over several weeks [21], suggesting that additional iodine exposure may have contributed to the higher TSH response in the Sb-Ac-NaCaThIo implants. By 12 weeks, TSH levels stabilized without significant changes, and at 13 weeks they decreased in both implants, indicating recovery. These findings suggest that while iodine loading on the implant surface transiently elevates TSH, it does not exert long-term adverse effects on thyroid function.

Facultative anaerobes alone or facultative and obligate anaerobes have been reported to be isolated from dental abscesses, with the most common facultative anaerobes being the Viridans group streptococci and the anginosus group streptococci [50]. Since *S. mitis* is a representative species of the Viridans group streptococci [51], it was selected as a clinically relevant bacterium for the present study.

The inter-rater reliability of the inflammation scores assessed by three independent evaluators was Fleiss’ κ = 0.53, indicating moderate agreement and supporting the robustness of the histological evaluation. As shown in Figure 14, inflammatory cell infiltration peaked at 2 weeks and subsequently decreased at 4 weeks. This temporal pattern is consistent with the definition of postoperative infection in the oral cavity, which typically appears ≥10 days after surgery [52]. The marked increase at 2 weeks is therefore biologically plausible and likely reflects the combined influence of surgical invasion and bacterial infection. Importantly, at this 2-week time point, the (1) Ac-NaCaThIo + *S. mitis* group demonstrated lower infiltration scores compared with the (2) no treatment + *S. mitis* group. This suggests that the antibacterial activity of iodine incorporated into the Ac-NaCaThIo-treated titanium plates contributed to suppression of inflammatory cell infiltration. Although a causal relationship cannot be fully established within the limitations of this animal model, the consistent reduction in inflammation observed in the (1) Ac-NaCaThIo + *S. mitis* group supports the interpretation that this surface treatment has potential anti-inflammatory benefits. By 4 weeks, inflammatory cell infiltration was markedly reduced across all groups, which can reasonably be attributed to wound healing and the resolution of bacterial challenge over time.

This study has several limitations. Implant stability was assessed using ISQ, but biomechanical tests such as removal torque were not included. Furthermore, although histomorphometric image analysis was performed on tissue sections, micro-CT analyses were not conducted, and therefore three-dimensional bone evaluation was not possible. Incorporating these approaches would provide additional evidence to further support the findings of this study.

## 5. Conclusions

Acid etching followed by NaCaThIo-treated implants had micro/nano-scale structures on the implant surfaces. Although non-inferiority in ISQ compared with commercial implants could not be demonstrated, histological analyses revealed favorable bone formation and bone-to-implant contact. Furthermore, the iodine loaded on the implants had no long-term effect in vivo. In addition, surface-treated titanium tended to exhibit anti-inflammatory properties against *S. mitis*. Therefore, although further investigations are required, this surface treatment method is expected to be applied to dental implants with anti-inflammatory properties for jawbones with insufficient height and to artificial bones with anti-inflammatory properties that require good bone-bonding strength for bone defects, suggesting its potential applicability.

## Figures and Tables

**Table 1:** Procedures of the NaCaThIo treatment.

	1st Treatment	2nd Treatment	3rd Treatment	4th Treatment
NaCaThIo treatment	5 mol·L^−1^ NaOH	100 mmol·L^−1^ CaCl_2_	600 °C, 1 h	10 mmol·L^−1^ ICl_3_
	60 °C, 24 h	40 °C, 24 h		80 °C, 24 h

**Table 2:** Surface treatment methods and procedures for the titanium disks and the dental implants.

Substrate	1st Treatment	2nd Treatment	3rd Treatment
Sb-Ac-NaCaThIo titanium disks	Sandblast treatment	Oxalic acid etching treatment	NaCaThIo treatment
Sb-Ac titanium disks	Sandblast treatment	Oxalic acid etching treatment	-
Sb-Ac-NaCaThIo implants	Sandblast treatment	Oxalic acid etching treatment	NaCaThIo treatment
Sb-Ac implants	Sandblast treatment	Oxalic acid etching treatment	-

“-”: No treatment.

**Table 3:** Surface treatment method and procedure applied to the titanium plate.

Substrate	1st Treatment	2nd Treatment
Ac-NaCaThIo titanium plate	Mixed acid etching treatment	NaCaThIo treatment
No treated titanium plate	-	-

“-”: No treatment.

**Table 4:** EDX quantitative analysis results of the placed titanium disk surface.

Sample		Element (wt%)								
Surface Treatment	Immersion Period	O	Ti	Ca	P	Na	Mg	I	Al	Si
Sb-Ac-NaCaThIo	Before immersion	66.7	30.2	2.6	-	-	-	0.8	0.3	0.4
Sb-Ac-NaCaThIo	3 d	63.6	17.0	11.3	6.8	0.2	0.5	U.D.	U.D.	0.4
Sb-Ac-NaCaThIo	7 d	63.7	6.3	16.8	11.7	0.4	0.5	U.D.	U.D.	0.4
Sb-Ac	Before immersion	4.5	94.9	U.D.	-	-	-	U.D.	0.2	0.2
Sb-Ac	3 d	5.9	93.2	U.D.	U.D.	U.D.	U.D.	U.D.	0.2	0.1
Sb-Ac	7 d	5.7	93.6	U.D.	U.D.	U.D.	U.D.	U.D.	0.1	0.2

U.D.: No peak detection; “-”: Not measured.

## Data Availability

The data presented in this study are available upon request from the corresponding author due to institutional regulations and ethical restrictions.

## Abbreviations

The following abbreviations are used in this manuscript:

SBF	Simulated Body Fluid
FE-SEM	Field Emission Scanning Electron Microscope
EDX	Energy-dispersive X-ray spectroscopy
ISQ	Implant Stability Quotient
RFA	Resonance Frequency Analysis
BAO	Bone Area Occupancy
BIC	Bone-Implant Contact Ratio
TSH	Thyroid-Stimulating Hormone
